# A140 ECTOPIC SPLEEN PRESENTING AS A GASTRIC SUBMUCOSAL LESION

**DOI:** 10.1093/jcag/gwac036.140

**Published:** 2023-03-07

**Authors:** I Balubaid, A Almudaires, M Sey

**Affiliations:** Gastroenterology, Western University, London, Canada

## Abstract

**Background:**

Accessory spleen is ectopic splenic tissue which can be either congenital or acquired. It is usually an asymptomatic incidental finding. In the context of known malignancy, accessory spleen can be mistaken for metastasis.

**Purpose:**

Here, we share a case of ectopic spleen presenting as a gastric subepithelial lesion post splenectomy. Rare cases have been reported in the literature.

**Method:**

Case Report and Literature Review

**Result(s):**

**Case:**

A middle age male with history of splenic rupture secondary to a motor vehicle accident and metastatic renal cancer was found to have a gastric subepithelial lesion on endoscopy. Endoscopic ultrasound (EUS) revealed a subepithelial mass coming off the submucosa or muscularis mucosa of the gastric wall with intact muscularis propria. EUS-guided core biopsy was performed and demonstrated splenic tissue

**Conclusion(s):**

Splenic tissue should be considered in the differential diagnosis when assessing gastric subepithelial tumors even in the context of previous trauma to the spleen. In addition, EUS-guided core biopsy may be required to rule out metastatic disease.

**Please acknowledge all funding agencies by checking the applicable boxes below:**

None

**Disclosure of Interest:**

None Declared

ESOPHAGEAL, GASTRIC & DUODENAL DISORDERS

